# Cerebrospinal Fluid Levels of sAPP**α** and sAPP**β** in Lewy Body and Alzheimer's Disease: Clinical and Neurochemical Correlates

**DOI:** 10.4061/2011/495025

**Published:** 2011-09-28

**Authors:** Ezra Mulugeta, Elisabet Londos, Oskar Hansson, Clive Ballard, Ragnhild Skogseth, Lennart Minthon, Kaj Blennow, Henrik Zetterberg, Dag Aarsland

**Affiliations:** ^1^Centre for Age-Related Medicine, Stavanger University Hospital, 4068 Stavanger, Norway; ^2^Wolfson Centre for Age-Related Diseases, King's College London, London SE1 1UL, UK; ^3^Research Unit, Department of Clinical Sciences, Malmö, University of Lund, Lund, Norway; ^4^Department of Psychiatry and Neurochemistry, Institute of Neuroscience and Physiology, The Sahlgrenska Academy, University of Gothenburg, Molndal, Gothenburg, Sweden; ^5^NVS Department, KI Alzheimer Disease Research Center, Karolinska Institutet, Novum, 14186 Stockholm, Sweden; ^6^Institute of Clinical Medicine, Akershus University Hospital, University of Oslo, Oslo, Norway

## Abstract

We measured cerebrospinal fluid (CSF) levels of the soluble isoforms of amyloid precursor protein (APP; sAPP**α** sAPP**β**) and other CSF biomarkers in 107 patients with Alzheimer's disease (AD), dementia with Lewy body dementia (DLB), Parkinson's disease dementia (PDD), and normal controls (NC) using commercial kits. DLB and PDD were combined in a Lewy body dementia group (LBD). No differences were observed in sAPP**α** and sAPP**β** levels between the groups. Significant correlations were observed between sAPP**α** and sAPP**β** and between sAPP**β** and Mini-Mental State Examination scores in the total group analysis as well as when LBD and AD groups were analyzed separately. sAPP**α** and sAPP**β** levels correlated with A**β**38, A**β**40, A**β**42, and Tau in the LBD group. In AD, sAPP**α** correlated with p-Tau and sAPP**β** with A**β**40. The differential association between sAPP**α** and sAPP**β** with A**β** and Tau species between LBD and AD groups suggests a possible relationship with the underlying pathologies in LBD and AD.

## 1. Introduction

Cerebrospinal fluid (CSF) levels of amyloid beta peptides (A*β*) and Tau proteins have shown good accuracy in distinguishing early Alzheimer's disease (AD) from healthy elderly controls [1], but studies from dementia with Lewy bodies (DLB) and Parkinson's disease dementia (PDD) are scarce. 

The A*β* peptides originate from the transmembranous amyloid precursor protein (APP) after sequential cleavage by proteases known as *β*- and *γ*-secretases [2]. Even though APP is ubiquitously expressed, its physiological role is not yet known [3]. Some studies suggest a role of APP in neuronal survival and migration, neurite outgrowth, synaptic plasticity, and cell adhesion [4]. Cleavage of APP by *α*-secretase generates a soluble protein known as sApp*α*, which precludes the formation of the toxic 42 amino acid long A*β* peptide (A*β*42) [3, 4]. 

The toxic A*β*42 peptide as well as the shorter nontoxic and more abundant A*β* variants (A*β*38 and A*β*40) are generated following the amyloidogenic pathway which starts by extra cellular cleavage of APP by the enzymes BACE and *γ*-secretase and results in the production of soluble sAPP*β*. Thus sAPP*α* and sAPP*β* are secreted APP fragments. However, the physiological role of these peptides is not well established. Production of sAPP*α* increases in response to electrical activity and activation of muscarinic acetylcholine receptors [5] as well as increasing synaptic and neurotrophic activities [6]. Until recently the physiological role of sAPP*β* was associated mainly with the amyloidogenic pathway in AD. However, a recent investigation by Li et al. presented a new possible role for the soluble sAPP*β* as a transcriptional enhancer for some proteins that seem to be involved in the APP cascade [7]. 

Inconsistent results have been reported among the few studies exploring CSF levels of sAPP*α* and sAPP*β* as potential biomarkers for AD. Lewczuk and coworkers recently reported significant increases of both sAPP*α* and sAPP*β* in neurochemically verified AD patients in comparison to a group consisting of nonneurochemically verified dementia cases [8], and Gabelle et al. reported similar findings [9]. In contrast, Zetterberg et al. detected no significant differences between AD patients and cognitively healthy controls [10], and an earlier study by Olsson et al. [11] found no significant differences in sAPP*α* or sAPP*β* levels between healthy controls and patients with sporadic AD. However, the level of sAPP*β* was elevated in patients with mild cognitive impairment compared to controls [11]. 

Neuropathological [12] and CSF [13] studies have shown that pathological processing of amyloid occurs also in DLB and PDD, which represent 15–20% of the dementia population [14]. Thus, it is possible that these proteins may somehow be involved in the pathogenesis of these diseases as well as in AD. To our knowledge, the levels of sAPP*α* and sAPP*β* have not yet been investigated in patients with DLB and PDD [15]. In this study our objective was to examine the CSF level of sAPP*α* and sAPP*β* in patients with AD, DLB, PDD, and normal controls (NC). A second objective was to explore clinical and neurochemical correlates of CSF sAPP*α* and sAPP*β* levels, to assess their relationship with disease severity as a measure of underlying brain pathology.

## 2. Methods

### 2.1. Subjects

Patients were drawn from two cohorts in Scandinavia: the DemWest study recruited patients with mild dementia from all geriatric, psychiatric and neurology outpatient clinics in Rogaland and Hordaland counties in Western Norway between 2005–2007 [14]. Out of the total 225 patients, 55 individuals consented for lumbar puncture and CSF collection, and those with AD (*n* = 31), DLB (*n* = 9), or PDD (*n* = 3) were included in the study. Twelve elderly subjects without a subjective cognitive impairment or known brain disease and MMSE score of >25 who consented to lumbar puncture at the Stavanger University Hospital during orthopaedic surgery or neurologic outpatient assessment were included as nondemented controls (NC). A cohort from the Neuropsychiatric Clinic, Skåne University Hospital in southern Sweden consisted of patients with PDD (*n* = 18) and probable DLB (*n* = 15) who participated in a clinical trial of memantine [16], and of 19 patients with AD from the Outpatient Memory Clinic.

Diagnostic procedures are described in detail elsewhere [14, 16, 17]. In brief, all patients were diagnosed according to clinical consensus criteria for probable DLB [18], probable AD [19] or PDD [20] after a detailed clinical assessment by a registered specialist in psychiatry, neurology, or geriatric medicine, using standardised and validated questionnaire for functional consequences of cognitive impairment [21] and neuropsychological testing.

### 2.2. Preanalytical Treatment of CSF

Lumbar puncture (LP) was performed in the L3-L4 or L4-L5 interspace, and CSF sampling was performed in all cases between 7–10 am in order to minimize diurnal variation of the level of CSF A*β* [22]. The first 3–4 mLs of the CSF were dedicated for routine analyses for assessment of relevant CSF abnormalities, and immediately sent on ice to the routine laboratory where cell counts and measurements of glucose and protein were performed. Study samples were collected in separate polypropylene tubes, and centrifuged at 2000 ×g, 4°C for 10 min to get rid of cell debris and other insoluble materials. Following centrifugation, samples were aliquoted and immediately frozen at −80°C until analyses were performed. Samples from the DemWest study, Stavanger, were originally stored in larger volumes, thus the portions of samples used in this study were aliquots derived from samples frozen and thawed (on ice) once.

### 2.3. sAPP*α* and sAPP*β* Assay in Patients CSF by Electrochemiluminescence Assay

All CSF analyses were performed randomized and in duplicate the same day by E. Mulugeta, blinded to clinical information. CSF levels of sAPP*α* and sAPP*β* were determined using the multiplex assay (Human A*β* peptide Ultra-Sensitive Kits) developed by Meso Scale Discovery, Gaithersburg, Maryland, USA. The assay uses the 6E10 antibody to capture sAPP*α* and a neoepitope-specific antibody to capture sAPP*β*, combined with the sulfotagged antibody P2-1 (reacting with the N-terminal domain of APP) for detection. The assay technology is based on MULTI-ARRAY technology combining electrochemiluminescence detection and patterned arrays offering combination of sensitivity and dynamic range. The assay was performed on CSF samples from patients and control subjects were diluted 1 : 4 and standards of sAPP*α* and sAPP*β* (in concentration range 7.8–500 ng/mL) were prepared and finally all samples and standards were run in duplicate, and assay procedures were applied as recommended in the manufacturer's instructions. Finally electrochemiluminescence signals were captured from photo detectors, and signals were processed using the software Discovery Workbench 3 (MSD). Standard concentrations of each analyte were plotted in a 4-parameter logistic fit curve and the concentration values of the unknown samples were computed.

### 2.4. Analyses of A*β* Peptides and Tau Protein

The analytical methods for triplex A*β*38, A*β*40, A*β*42, as well as Tau and P-Tau assays have been described previously [13]. P-Tau levels were available only for 31 of the 50 AD cases. 

### 2.5. Statistics and Analytical Procedures

In the final analyses a total of 107 subjects were included: 50 AD, 45 LBD, and 12 NC. Demographics of the patients and control subjects are shown in Table 1. The groups did not differ by age. DLB and PDD groups displayed significant difference in disease duration, as expected due to the relatively long duration with pure motor symptoms in PD before dementia, but not on other demographic data, clinical, or biochemical values (Tables 1 and 2). They were therefore combined in a Lewy-body disease group (LBD) for the subsequent analyses. Mean MMSE score between the AD group and the LBD group did not differ significantly. Values of CSF markers were expressed as absolute (ng/mL) for sAPP*α* and sAPP*β* and (pg/mL) for A*β*38, A*β*40, A*β*42, and Tau. Between-group comparisons were performed by using chi-square tests or Kruskall-Wallis followed by Mann Whitney U test. Correlations between levels of CSF analytes and demographic and clinical features were performed using Spearman correlation for nonparametric analyses. Correlations were considered significant if they reached the 0.05 level (2-tailed) and highly significant at the 0.01 level (2-tailed).

## 3. Results

The standard ranges for sAPP*α* and sAPP*β* were 7.81–500 ng/mL. The lower limit of detection for sAPP*α* was (3.88 ng/mL) and plate-to-plate variation on lower range of detection was 14%. For sAPP*β* the lower range of detection was (2.25 ng/mL) and plate to plate variation on the lower range of detection was 12%. To determine inter- and intraassay variations we have chosen to analyze one CSF sample on each occasion/plate as a run control. The interassay variability (same sample analyzed on different plates *n* = 6) for sAPP*α* and sAPP*β* was 13% and 12%, respectively. The analytical performance of the A*β* and Tau measurements have been described previously[13]. 

### 3.1. Levels of sAPP*α* and sAPP*β*

For the total group, mean (SD) of sAPP*α* was 706 (228) ng/mL, and sAPP*β* was 382 (89). There were no significant differences observed in the levels of sAPP*α* (*P* = 0.68) and sAPP*β* (*P* = 0.59) between the groups (Table 2).

### 3.2. Correlations

The correlations between sAPP*α* and sAPP*β* levels and MMSE scores as well as levels of A*β* peptides and Tau are shown in Table 3. Highly significant correlations were found between sAPP*α* and sAPP*β* in all groups (Table 3). MMSE correlated significantly with both sAPP*α* and sAPP*β* in the total group, but was significantly associated only with sAPP*β* in LBD and AD groups. However, the correlation factors of sAPP*α* in the patient groups were rather similar to the control group, suggesting that the lack of significance is related to the smaller sample size in the individual groups. In the LBD group significant correlations were found between sAPP*α* and sAPP*β* with all A*β* species and T-Tau, but not P-tau. In the AD group a significant association was observed only between sAPP*β* and A*β*40 and between sAPP*α* and P-tau.

## 4. Discussion

This study examines the CSF levels of sAPP*α* and sAPP*β* in patients with LBD and AD. We found no difference between AD and LBD patients and normal controls using multiplex immunoassay method. Another finding was the highly significant correlations between sAPP*α* and sAPP*β* in all groups. A key finding was that MMSE scores were associated with sAPP*β*, but less so with sAPP*α*, in AD and LBD groups. Finally, there were associations between both sAPPs and all A*β* species and T-Tau in the LBD group whereas in the AD group there were association between sAPP*β* and A*β*40 and between sAPP*α* and P-tau.

Following the assumption of alternative APP processing in either the amyloidogenic or nonamyloidogenic way, one would expect a reverse correlation of sApp*α* to sAPP*β*. However, we found a positive correlation, which is consistent with previous studies [8–10]. The reason why the CSF levels of these APP isoforms correlate tightly is unknown, but may suggest that the expression of APP isoforms are regulated by similar stimuli and pathways, and thus that they have similar functions. Alternatively, only the secretion of the APP isoforms into the extracellular space and CSF are coregulated. Another possible explanation could be the presence of another factor that regulates the activity of the enzymes involved in the APP processing. 

## 5. Association of sAPPs with Dementia Severity and Other CSF Markers

The association between MMSE and sAPP might suggest that secretion of sAPP*β* into the CSF is associated with less severe pathology as a result of less A*β*42 deposition and plaque formation and might represent less severity and perhaps a potential protective mechanism. The potentially neuroprotective properties of sAPP*β* discussed by Li et al. [7] may overbalance the neurotoxic potential of A*β*42. In cell studies it has been suggested that APP fragments regulate behavioral learning and memory [23–27]. The association between MMSE score and the sAPP*β* both in AD and DLB patients are to some extent consistent with findings in cell models suggesting that cognitive function in AD or LBD may be are affected by altered levels and activity of the different APP fragments. 

Whereas our findings of no difference between the diagnostic groups are in line with some previous studies, they contrast with some others studies [8, 9]. Although similar methods for measuring sAPP were used, possible explanations for inconsistent finding are that in the two previous studies, the non-AD group included mainly patients with frontotemporal dementia.

We speculate that sAPP*β* release somehow stimulates brain plasticity, which might correlate with higher MMSE in all disease groups and Tau levels in the upper normal range in LBD (CSF Tau levels in the normal range might be a plasticity marker) [28]. However, it should be emphasized that these data are in need of replication in other studies and larger cohorts.

The differential association between sAPP*α* and sAPP*β* with the classical AD markers amyloid and Tau pathology in AD compared to LBD suggests different pathological mechanisms. This might be related to the more severe amyloid and tau pathologies in AD compared to DLB and PD, or it is possible that *α*-synuclein pathology in some way affects the association between sAPP*α* and sAPP*β* with the development of AD pathology. 

In AD, the A*β* species most likely reflect the primary and fundamental pathologic events. Specifically, amyloid deposition is correlated to low CSF levels of A*β*42 and its ratio to A*β*40/A*β*38. T-Tau is a marker of axonal neurodegeneration whereas P-Tau is a marker of Tau hyperphosphorylation and neurofibrillary tangle formation [1]. Thus, the observed correlation of both sAPPs with T-Tau, especially in the LBD group, suggests that the sAPPs are involved in axonal processing that in turn may indicate ongoing axonal damage.

## 6. Methodological Limitations

Methodological limitations which might influence the findings include the relatively small number of patients with low statistical power. Also, this was an exploratory study, and we did not adjust for the large number of comparisons when correlation analyses were performed. Our findings should therefore be interpreted with caution. 

The controls were not assessed with standard neuropsychological tests. Although they were without subjective cognitive complaints, a subtle cognitive impairment indicating very early AD cannot be completely excluded. Finally, patients were recruited from two different centres. However, the diagnostic procedures at the two centres were harmonized through several meetings and the use of the same protocols, but no formal reliability analyses were performed to assess the consistency as the number of patients in individual patient group was not so large.

## 7. Conclusions

Although the CSF levels of sAPP*α* and sAPP*β* did not differ between AD, DLB, and NC, there were disease-specific differences in their associations with A*β* and Tau species. Further studies are needed to explore this, and to explore how these peptides are associated with dementia severity.

## Figures and Tables

**Table 1:** Demographic and clinical characteristics of the diagnostic groups.

Diagnosis groups	NC (*n* = 12)	AD (*n* = 50)	DLB (*n* = 24)	PDD (*n* = 21)	*P*
Gender (F/M)	7/5	30/20	7/17	7/14	0.034
Age, y	74.1 (8.3)	74.4 (8.1)	74.0 (7.1)	73.6 (7.0)	—
MMSE	28.6 (0.9)	21.1 (4.9)	21.8 (4.0)	20.4 (4.9)	0.000^(1)^
Disease duration, y	Na	2.2 (1.3)	4.1 (3.1)	7.0 (4)	0.0002^(2)^
Education, y	10.2 (3.0)	10.1 (.3.) (*n* = 27)	9.3 (2.4)	9.3 (2.8)	0.8

NC: normal controls, AD: Alzheimer's disease, DLB: dementia with Lewy bodies, PDD: Parkinson's disease dementia; Na: not applicable.

Numbers represent mean (standard deviation) or number of patients.

^(1)^Significant difference between NC and the disease groups. No significant difference between disease groups.

^(2)^Significant difference between PDD and the two other disease groups. No significant difference between DLB and AD.

**Table 2:** Comparison of CSF values between groups.

	NC (*n* = 12)	AD (*n* = 50)	DLB (*n* = 24)	PDD (*n* = 21)	P*
A*β*38 [pg/mL] (SD)	785 (399)	814 (468)	384 (277)	366 (209)	0.000
A*β*40 [pg/mL] (SD)	7835 (3152)	5297 (1813)	5833 (3033)	262 (206)	0.006
A*β*42 [pg/mL] (SD)	479 (282)	275 (121)	262 (206)	264 (115)	0.001
Tau [pg/mL] (SD)	269 (119)	600 (291)	297 (109)	308 (201)	0.000
P-Tau [pg/mL] (SD)	58 (24) *n* = 7	89 (38) *n* = 39	63 (26) *n* = 20	66 (28) *n* = 19	0.008
sApp*α* [ng/mL] (SD)	744 (259)	716 (209)	646 (212)	728 (271)	0.677
sApp*β* [ng/mL] (SD)	413 (84)	380 (86)	364 (88)	387 (99)	0.585

*Kruskal-Wallis test.

**Table 3:** Correlations between CSF analytes and MMSE score.

	MMSE	A*β*38	A*β*40	A*β*42	Tau	P-Tau^†^	sApp*β*
All diagnoses together (total group *n* = 107)							
sApp*α*	0.206*	0.337**	0.291**	0.220*	0.285**	0.411**	0.876**
sApp*β*	0.384**	0.295**	0.549**	0.233*	0.074	0.329**	—
Tau	−0.241*	0.718**	0.001	0.193	—	—	0.113
P-Tau	0.133	0.488**	0.259*	−0.103	—	—	0.329**

NC (*n* = 12)							
sApp*α*	−0.059	0.622*	0.497	0.643*	0.286	0.357 Obs *n* = 7	0.865**
sApp*β*	0.018	0.658*	0.616*	0.515	0.214	0.429 *n* = 7	—
Tau	0.386	0.464	0.393	0.429	—	—	0.214
P-Tau	−0.540	0.964**	0.821*	0.857**	—	—	0.429

AD (*n* = 50)							
sApp*α*	0.179	0.071	0.009	−0.093	0.146	0.570** *n* = 31	0.811**
sApp*β*	0.422**	−0.123	0.395**	−0.168	−0.154	0.340 *n* = 31	—
Tau	−0.579**	0.723**	−0.371**	0.330∗	NR	NR	−0.154
P-Tau	0.107	0.454*	0.148	−0.132	—	—	0.340

LBD (*n* = 45)							
sApp*α*	0.289	0.537**	0.430**	0.417**	0.428**	0.252	0.927**
sApp*β*	0.371*	0.663**	0.608**	0.498**	0.446**	0.274	—
Tau	0.234	0.675**	0.629**	0.362*	—	—	0.446*
P-Tau	0.255	0.510**	0.431**	0.134	—	—	0.274

DLB (*n* = 24)							
sApp*α*	0.297	0.481*	0.461*	0.359 *P* = 0.09	0.377	0.408	0.930**
sApp*β*	0.314	0.527**	0.600**	0.401 *P* = 0.058	0.274	0.299	—
Tau	0.156	0.581**	0.471*	0.185	—	—	0.274
P-Tau	0.422	0.421	0.211	−0.060	—	—	0.299

PDD (*n* = 21)							
sApp*α*	0.300	0.593**	0.456*	0.542*	0.483*	0.185	0.902**
sApp*β*	—	0.791**	0.641**	0.644**	0.618**	0.312	—
Tau	0.249	0.809**	0.850**	0.565**	NR	NR	0.618**
P-Tau	0.110	0.624**	0.792**	0.315	NR	NR	0.312

Spearman correlation. Numbers represent *ρ* values. *Correlation is significant at the 0.05 level, **correlation is significant at the 0.01 level. NR = no relevance. ^†^P-Tau: incomplete data (total *n* = 78).
